# Human Infant Faces Provoke Implicit Positive Affective Responses in Parents and Non-Parents Alike

**DOI:** 10.1371/journal.pone.0080379

**Published:** 2013-11-25

**Authors:** Vincenzo Paolo Senese, Simona De Falco, Marc H. Bornstein, Andrea Caria, Simona Buffolino, Paola Venuti

**Affiliations:** 1 Psychometric Laboratory, Department of Psychology, Second University of Naples, Caserta, Italy; 2 Department of Psychology and Cognitive Sciences, University of Trento, Trento, Italy; 3 Eunice Kennedy Shriver National Institute of Child Health and Human Development, National Institutes of Health, U.S. Department of Health and Human Services, Bethesda, Maryland, United States of America; 4 Institute of Medical Psychology and Behavioral Neurobiology Eberhard Karls, University of Tübingen, Tübingen, Germany; University G. d'Annunzio, Italy

## Abstract

Human infants' complete dependence on adult caregiving suggests that mechanisms associated with adult responsiveness to infant cues might be deeply embedded in the brain. Behavioural and neuroimaging research has produced converging evidence for adults' positive disposition to infant cues, but these studies have not investigated directly the valence of adults' reactions, how they are moderated by biological and social factors, and if they relate to child caregiving. This study examines implicit affective responses of 90 adults toward faces of human and non-human (cats and dogs) infants and adults. Implicit reactions were assessed with Single Category Implicit Association Tests, and reports of childrearing behaviours were assessed by the Parental Style Questionnaire. The results showed that human infant faces represent highly biologically relevant stimuli that capture attention and are implicitly associated with positive emotions. This reaction holds independent of gender and parenthood status and is associated with ideal parenting behaviors.

## Introduction

Adults tend to respond promptly and appropriately to babies from the first hours after birth [1]–[5]. This propensity to respond to infants depends on processing infant cues of various sorts, from smells to sounds to sights [6]. Notably, infant faces have a specific configuration that appears to elicit solicitous caregiving. The ethologist Konrad Lorenz [7]–[8] famously identified a constellation of morphological characteristics that distinguish infant from adult faces. Infant physiognomies include such features as a head large in proportion to the body, a protruding forehead relative to the size of the rest of the face, large eyes set low in the head, and round protruding cheeks [9]–[10]. Baby faces elicit willingness to approach, to smile, and to communicate [11]–[12]. Moreover, adults' sensitive responsiveness fosters cognitive, emotional, and social development in the child [13]–[18]. By contrast, adults who show low warmth or insensitivity [19]–[20], rejection [19],[21]–[22], or authoritarian [23] or disengaged caregiving [21],[24] impede positive child development.

Some variables that affect the adult-child relationship and child caregiving are biological (e.g., gender, motherhood status), and others are psychological characteristics of adults (e.g., attitudes), of infants (e.g., temperament), and of the family (e.g., SES) [25]–[30]. As regards individual-difference factors, the literature shows that attitudes toward children influence how caregivers interact with children [27],[31]. Attitudes have often been considered to be good predictors of parenting behaviours because they indicate the valence of the emotional climate in which children and parents interact. Until now, however, most research has focused on adults' self- expressed thoughts and feelings about their child-related behaviours [1],[33].

In contrast with this general orientation, studies from cognitive, social, and developmental science [34]–[36] have revealed that much cognition occurs outside of conscious awareness [37]–[39]. To measure cognitions that are normally inaccessible to conscious introspection, indirect measures have been developed. These measures have in common that cognitions are estimated without seeking direct verbal report [36],[40], thus reducing the impact of deliberative processes on them. Among such indirect measures, the Implicit Association Test (IAT) [41] is the one most widely used and best validated. The IAT is a behavioural paradigm designed to evaluate comparative implicit reactions toward two contrasting target objects and to define their affective valence or implicit attitude. Studies using the IAT have shown that even complex behaviours (e.g., voting or deviant sexual activity) are attributed valence in implicit reactions [42]–[44]. Through their meta-analysis of the predictive validity of the IAT scores, Greenwald et al. [43] found that (a) IAT and self-report measures have mutual incremental validity in predicting behavior; (b) IAT measures have greater predictive validity than self-reports for criterion measures involving social behavior; and (c) IAT measures predict both controlled and spontaneous actions. Additional research has shown that automatic or implicit processes are especially influential in driving behaviours when individuals are under stress or when they have little time to choose among appropriate behaviours [45].

Normally, decisions associated with parenting infants need to be fast. Moreover, the long evolutionary history of infant dependency on adult caregiving suggests that mechanisms associated with adult responsiveness to infants ought to be neurally supported. Adult responsiveness to human infant cues should therefore reflect allocation of increased attention and readiness to respond. Such responsiveness would possess adaptive value in favoring parent-child bonding as well as offspring and species survival and well-being [46]–[47]. Extant research confirms that adults have rapid, specific, and brain-based reactions to baby faces. Brosch, Sander, and Scherer [48], for example, examined the hypothesis that the baby schema facilitates attention. They compared responses of adults to pictures of faces depicting babies, adults, and non-human mammal infants and adults (cats and dogs) in a dot probe task. Results showed specific attentional capture by pictures of human infants. Notably, no gender differences were found for the attentional bias associated with the baby schema; females and males were equivalently sensitive.

The presence of specific reactions to (unfamiliar) baby cues has also been confirmed in neuroimaging research [49]. Studies that have investigated adult brain activity associated with the perception of baby faces have revealed that reward-related brain areas and the supplementary motor area react in specific ways to human infant facial cues, regardless of familiarity or kinship [10]–[11],[29],[50]–[51]. Among those studies, most germane here is one conducted by Kringelbach and colleagues [50] that examined adults' brain responses by means of magnetoencephalography (MEG). These investigators compared brain activation patterns of adults looking at unfamiliar infant and adult faces. They observed that the medial orbitofrontal cortex reacts to infant (not adult) faces rapidly (in about 130 ms) suggesting a possible role of this brain structure in (even unconsciously) guiding adult reactions. Their data also showed that parental status did not moderate these activation patterns.

Together, these studies converge to point to a generic, specific, and unconscious reaction in adults toward human baby faces, but they have failed to investigate directly whether adults' reactions have a positive or negative valence or how adults' reactions are moderated by biological and social factors. Moreover, in no case were relations between adults' neural reactions and their childrearing cognitions or practices investigated.

The present study aimed to examine adults' general unconscious disposition to human baby faces by means of a behavioural paradigm that allows the measurement of implicit affective reactions [52]. To evaluate adults' reactions we used the Single Category Implicit Association Test (SC-IAT) [53], a modification of the IAT that was designed to evaluate implicit associations of a single target concept. Similar to the IAT, the SC-IAT is fast to administer and allows evaluation of the affective valence of implicit reactions to a stimulus in a given context. In line with previous studies, and to determine whether adults' responses are specific for human infant faces [5],[11],[48], four SC-IATs were developed to evaluate adults' implicit reactions to faces of human and non-human (cats and dogs) infants and adults. We included non-human faces to assess whether expected positive implicit reactions are specific to human children or represent a more general inclination toward young offspring. To investigate how implicit reactions are moderated by biological and social factors, the effects of gender and parental status were examined. Finally, to determine whether adults' implicit reactions were associated with childrearing, participants completed a parental styles questionnaire.

We expected that human infant faces would trigger a specific response with a positive valence [1],[29],[54]. That is, we expected a main effect for stimulus with human infant faces being associated with positive attributes more than other faces. However, in accord with other studies, no gender or parental status differences were expected in adults' implicit reactions to infant faces [48],[50]. As regards the association between adults' implicit reactions and childrearing beliefs, in line with Greenwald et al. [43] we expected a moderate positive relation.

## Methods

### Ethics statement

The Local Research Ethics Committee, Department of Psychology, Second University of Naples, approved the study, and the written informed consent was provided by all participants.

### Sample

A total of 90 adults in 45 female-male couples participated (*M* age = 30.9 years, *SD* = 2.7, range 26 to 35 years). Twenty-one couples were parents and had an only child aged 15 to 30 months (*M* = 22.9 months; *SD* = 4.9), and 24 couples were non-parents. Parents and non-parents were *M* = 31.8 years (*SD* = 2.3) and *M* = 30.04 years (*SD* = 2.8), respectively, *t*(88) = −3.164, *p*<.01; their SES (computed by the four-factor Hollingshead Index) was *M* = 37.8 (*SD* = 4.2) and *M* = 35.0 (*SD* = 4.6), respectively, *t*(88) = −0.898, *p*<.372; and their educational levels varied from the middle school to college (*Mdn* =  high school). Although the mean age difference between parents and non-parents was only 1.8 years, to rule out effects of this age difference between groups, any main effects or interactions for parent/non-parent comparisons are followed up with controls for age.

### Materials and Procedures

Each participant was administered a sociodemographic form, four Single Category Implicit Association Tests, and the Parental Style Questionnaire. Testing sessions lasted about 25 min.

#### Sociodemographics

All participants completed a sociodemographic questionnaire.

#### Single Category Implicit Association Test (SC-IAT)

Following Karpinski and Steinman [53], four SC-IATs were adapted to evaluate automatic reactions to human and non-human infant and adult faces. The SC-IAT is a two-stage measure. In each stage, target words and pictures of a single target object are presented in random order. Participants are asked to classify words or pictures into the correct category as quickly as possible. Pictures refer to a single category, that is the target category, and words are distinguished as “good” and “bad” and have to be classified into a positive or negative category, respectively. In case of error, an “X” appeared at the centre of the screen. To reinforce speed of responding, a response window of 1500 ms following stimulus onset was applied for each stimulus. Each of the four classification tasks was repeated twice. The first time, good words and target object pictures were categorized on one response key, and bad words were categorized on a second key (positive condition). The second time, bad words and target object pictures were categorized on one response key, and good words were categorized on the second key (negative condition). The SC-IAT measure, also referred to as the IAT *effect*, derived from the comparison of latencies of responses in the two classification phases. The IAT effect is observed if response latency in the positive condition is slower than response latency in the negative condition. In other words, if participants are faster in categorizing stimuli when the target object is associated with the positive attribute in comparison to the condition that reflects negative−target object associations, then they are considered to have positive implicit attitudes towards the target. For each target object (faces in this study), and for each participant, the SC-IAT score was calculated using scoring algorithms related to *D* scores developed by Greenwald, Nosek, and Banaji [55]. This measure is computed by dividing the difference between means of RTs of the two classification tasks by the standard deviation of latencies of the two phases. Division of a difference between means by a standard deviation permits computation of an index that is similar to the well-known effect-size measure *d* [56]. IAT score values around 0 indicate no IAT effect; absolute values from 0.2 to 0.3 indicate a “slight” effect, values around 0.5 a “medium” effect, and values of about 0.8 to infinity a “large” effect [55]. In this study, positive values indicate that the target object was implicitly associated with the positive category. All the SC-IAT scores showed adequate reliability (*α*s>.70). For each SC-IAT we used ten different target words for the positive (positive, love, joy, beautiful, happy, paradise, present, pleasant, friend, smile) and negative categories (negative, hate, pain, bad, sad, hell, disaster, unpleasant, enemy, crying) and six variations of each of the four types of faces. Faces were selected from a database of coloured digital photographs [11]. Human faces were neutrally expressive, frontally oriented, and consisted of equal numbers of males and females (even though human infant faces had no cues to distinguish gender). Human adult faces portrayed people with a mean age of 25 years. Human infant faces portrayed infants with a mean age of about 6 months. Non-human pictures depicted equal numbers of frontally oriented cat and dog faces. Pictures were matched for size, brightness, color balance, and attractiveness. A total of 24 different faces (6 faces ×4 SC-IAT), six for each category (human and non-human infants and adults) were used. Stimuli were presented on a laptop screen by means of Inquisit 3.0.6 software and appeared approximately 35 cm from the participant's eyes. To avoid spurious effects due to sequence and order, the four SC-IATs were administered using a counterbalanced Latin-square design, and the association between target object and attribute of the first classification phase was counterbalanced across tasks.

#### Parental Style Questionnaire (PSQ)

The Parental Style Questionnaire [32],[57] is a 16-item self-report instrument designed to evaluate the frequency of mothers' and fathers' ways of interacting with infants and young children. PSQ items cluster into three parental style domains: social, didactic, and limiting setting (limit setting does not apply here). Each item describes a typical interaction between parent and child (e.g., “I promptly and appropriately respond to my child's expressed distress or discomfort.”, “I provide language learning opportunities for my child by labelling and describing qualities of objects, events, or activities, reading books, and so forth.” respectively, for Social and Didactic). In this study the Italian version of the scale was administered [58]–[59]. Parents were asked to report their actual interactions with their child and how they would ideally like to behave; non-parents were administered only the appropriately reworded ideal form. Each version had the same 16 items randomly ordered. Participants rated each item on a 5-point semantically anchored Likert-type scale ranging from 1 (*hardly at all*) to 5 (*all the time*). Items are scored so that high scores indicate more frequent Social and Didactic interactions.

### Data Analyses

To analyze main and interactive effects of adult gender and parent status on individual implicit affective reactions, we used a mixed factorial 4×2×2 ANOVA that treated faces as a 4-level within-subjects factor (human and non-human infant and adult), gender as a 2-level between-subjects factor (male and female), and parental status as a 2-level between-subjects factor (parent and non-parent). To test if effects were influenced by the parent versus non-parent age difference, we replicated the analyses using the age as between-subjects factor. In addition, because treating partners in a couple as separate cases violates an assumption of ANOVA, we replicated the analyses treating gender as within-subject factor. In both cases results did not change. For each stimulus, the *D* score (individual IAT effect) served as the dependent variable. Bonferroni correction was applied to analyze post hoc effects, and the magnitude of significant effects was indicated by partial eta squared (*η* ^2^_p_). Finally, for each effect statistical power was estimated post hoc by means of (GPower 3.0.10) software assuming an expected medium effect size. To analyze relations between implicit affective reactions toward baby faces and self-reported parental styles, correlation analysis was carried out.

## Results

**Figure 1:**
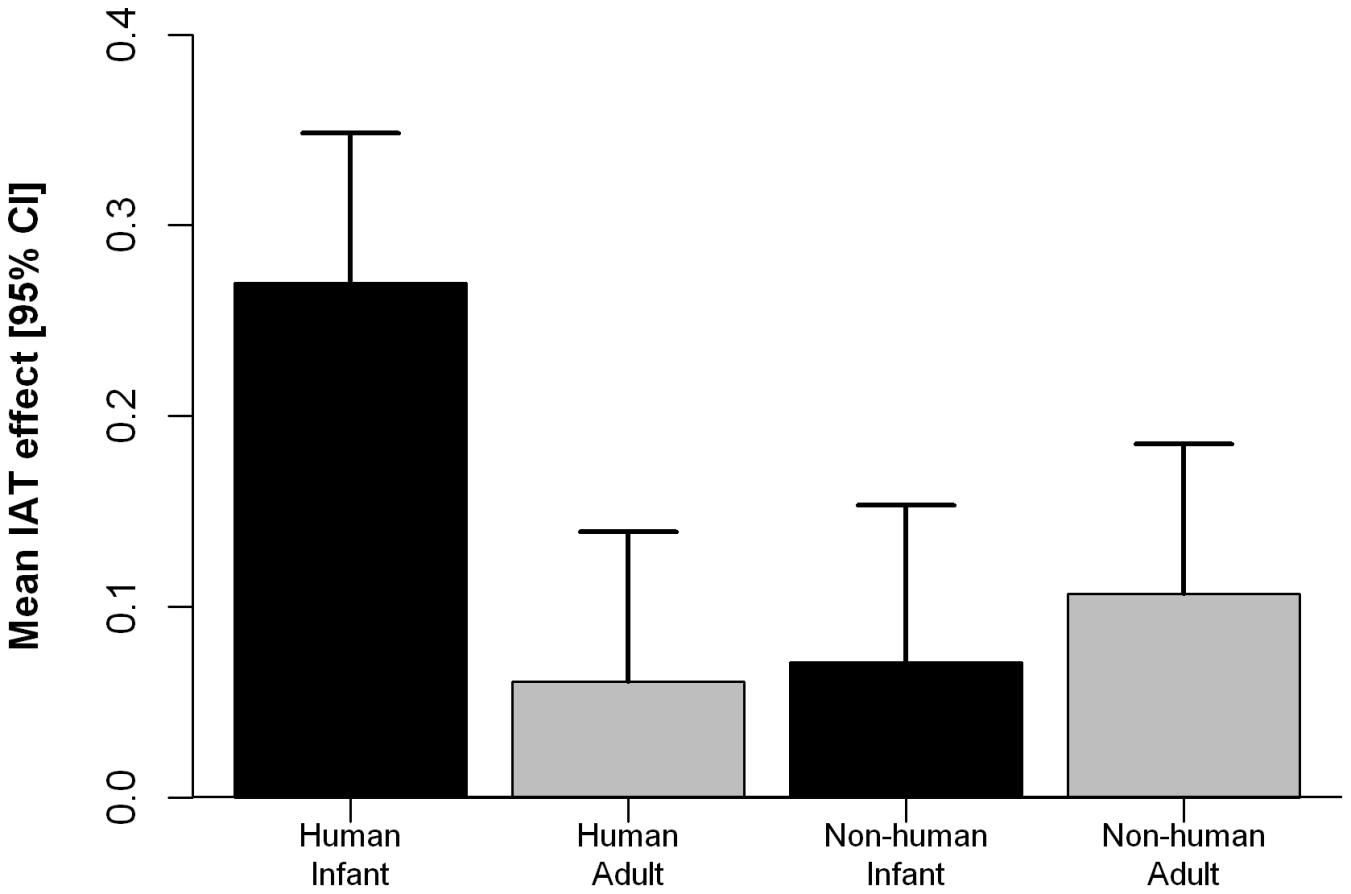
Mean IAT effect as a function of faces. Note. Error bars represent 95% CI.

The ANOVA on SC-IAT scores showed that implicit affective reactions were influenced only by faces, *F*(3,258) = 7.03, *p*<.001, *η^2^_p_* = .08, power = .999 (see Figure 1). No other significant main or interactive effects were observed.

Post hoc analysis revealed that human infant faces were associated with the positive dimension (*M* = 0.27, 95% CI [0.19, 0.35], *p*s<.01) more than human adult (*M* = 0.06, 95% CI [−0.02, 0.14]), animal infant (*M* = 0.07, 95% CI [−0.01, 0.15]), and animal adult (*M* = 0.11, 95% CI [0.03, 0.19]) faces. No differences were observed among the latter three face types. Moreover, the distribution of the SC-IAT scores showed a systematic specific association of the target stimuli with the positive dimension only for human infant faces (mean *D* value greater than .20), although a wide range of scores was observed at the individual level (range from −.55 to 1.39). In short, adults overall have a specific and significant, if small, implicit positive reaction to human baby cues that is not shared with human adults or infant or adult non-humans, but the variation among adults ranges from large positive implicit reactions to medium negative implicit reactions.

**Figure 2:**
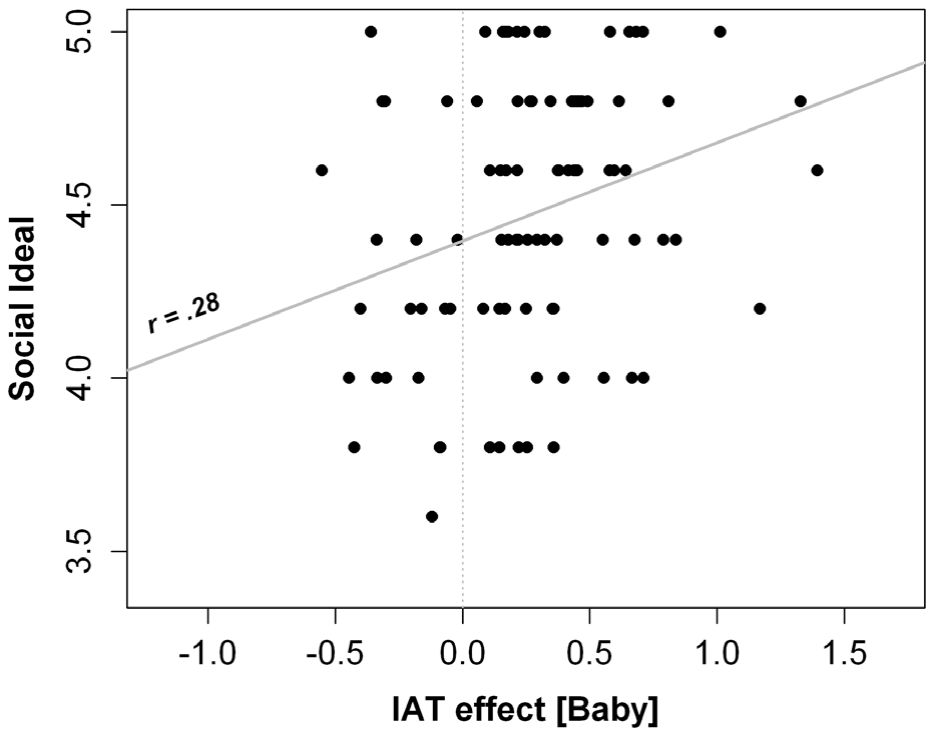
Scatter plot of the relation between the infant faces IAT effect and Social ideal style. Note. The solid line represents the linear relation model. The dashed line represents the neutral D score value.

Correlational analyses revealed positive associations between adults' automatic affective reactions toward human infant faces and their ideal parental styles. Specifically, SC-IAT scores related to human infant faces were significantly and positively associated with the ideal Social Scale, *r*(88) = .28, *p*<.01, *r* ^2^ = .08, power = .84 (see Figure 2). The more positive their implicit reaction to human infant faces, the more adults believe they ideally should interact socially with babies. These data confirm the “moderate” predictive validity of IAT measures discussed in the literature [43]. The correlation between infant SC-IAT scores and Didactic subscale was not significant, *r*(88) = .18, *p* = .09, *r* ^2^ = .03, power = .84. As regards actual parental style, evaluated only for parents (*n* = 42), no significant correlations were observed, *r*(40) = .08, *p* = .597, *r* ^2^ = .01, power = .51 and *r*(88) = −.12, *p* = .421, *r* ^2^ = .01, power = .51 respectively for Social and Didactic scales.

## Discussion

This study investigated implicit affective reactions of adults towards unfamiliar human and non-human infant and adult faces by means of a behavioural response paradigm (SC-IAT) that forces participants into an evaluative mode [52]. In the experimental procedure, faces were presented to females and males, parents and non-parents and their implicit affective reactions were measured. To investigate whether adults' reactions were specific to human children or represent a more general inclination toward infants, human and animal faces were contrasted. Consistent with other research, the results showed that human infant faces are strong cues associated with specific, implicit, and positive reactions [10]–[11],[48]. The results of this study show clearly that human baby faces, more than adult or non-human infant or adult faces, are associated with positive emotions when the association is measured with an IAT procedure. In accord with the literature in social psychology, adults show a unique (if small) positive implicit attitude toward even unfamiliar baby faces [36],[41]. In short, regardless of kinship, adults react positively to baby faces, and adults' responses are also specific to human infants.

Studies investigating individual factors affecting adults' reactions to infant cues have heretofore mainly focused on conscious processes, whereas the role of implicit processes in child-related behaviour regulation has been neglected [36],[45],[60]. To our knowledge this is the first study to evaluate adults' implicit reactions to unfamiliar baby cues. We found in a direct way that, when forced into an evaluative mode, adults express an implicit emotionally positive predisposition toward human infant faces. Our results accord with these of Brosch et al. [48], who showed specific and gender invariant attentional capture by pictures of human infants, and Kringelbach et al. [50], who observed that the medial orbitofrontal cortex reacts quickly and automatically to human infant faces. As here, in none of these studies did gender or parenthood status differentially moderate activation patterns.

Implicit measures often have direct relations with associated behaviours [40], though the mean predictive validity is moderate [43], and the relevance of implicit processes increases when individuals are under stress [45]. From this perspective, our results could indicate that positive actions toward babies are facilitated when adults have an emotionally positive and automatic reaction to baby cues. Caria et al. [11] showed enhanced involvement of the supplementary motor area as well as lateral premotor areas in specific response to human infant faces that they interpreted as supporting adults' “readiness” to interact with babies. It is important to note, however, that the implicit measure we used here revealed individual differences in unconscious reactions to baby faces of about 6-month-olds. It could be that positive implicit reactions vary with the age or attractiveness of infants [61]. For example, one study that investigated the development of explicit preferences for baby faces compared faces of infants from 3 to 24 months and found that participants evaluated the photographs of babies at the ages of 3 to 6 months to be cutest and young children and adults had similar preferences for baby faces, demonstrating the potency and universality of the baby schema [12]. To our knowledge no studies have investigated directly implicit preferences for baby faces of different ages. Future research is needed to determine how child age might affect adults' implicit reactions.

Some participants in this study did not show a specific preference toward human baby faces (*D* score values close to 0), whereas others showed a medium negative reaction (*D* score values greater than −.30). That is, around a significant positive mean, our results indicated individual variation in implicit dispositions toward babies. This variability could help to explain why adults behave in different ways towards infants and young children. It may be that implicit attitudes, more than explicit attitudes, regulate the degree of warmth or rejection that exists in the parent-child relationship [62]. In this connection, future research might explore SC-IAT scores in adults' at risk for child neglect or abuse. Young children elicit negative emotional responses in certain adults [63]–[64]. Because the relevance of automatic reactions for behaviour can be strengthened by the stress typically associated with infant caring, adults who display such negative reactions could be at higher risk for developing undesirable dynamics (such as abuse or neglect) that undermine the parent-child relationship and child development.

Implicit reactions toward human baby faces we studied might be biologically rooted. First, the SC-IAT paradigm calls on near automatic reactions that are likely deeply embedded in the adult brain [65]. Second, fMRI results show that adults' (even non-parents) processing of unfamiliar human infant faces, compared to carefully matched adult faces and non-human mammal infant and adult faces, activates brain systems associated with adults' preparation for communicative behavior as well as attachment and caregiving. Third, the implicit reactions we recorded were independent of gender and parenthood status. Although mothers are normally more engaged, available, and responsible for their young children than are fathers, and anthropological evidence indicates that mothers are the primary caregivers of young children in the vast majority of cultures [66], mothers and fathers normally show many similar patterns of interacting with infants, touching, looking at, vocalizing to, rocking, and kissing them equivalently [67]. Fathers can be as responsive to infants as mothers. Men and women exhibit similar stage-specific changes in hormone levels, including higher concentrations of prolactin and cortisol in the period just before the birth and lower postnatal concentrations of sex steroids (testosterone or estradiol); mothers and fathers are also often more similar than different in cognitive responses to children [68]–[69]. Likewise, parents and non-parents perceive infant cries similarly [70]. The literature on this topic seems to indicate that biological mechanisms that underlie responsiveness and a caring inclination toward young children transcend adults' gender and biological relationship to the baby. To understand brain functions or other deeply rooted responses, we need to understand the circumstances of the environment and social contingencies in which the nervous system evolved. The human social brain evolved in a situation (sometimes referred to as the environment of evolutionary adaptedness) where alloparenting was common, that is where many adults shared responsibility for infant care. Hrdy coined the term “cooperative breeder” to describe the way that multiple family members rear offspring in the group [71]. The literature on gender differences in responding to infants is marked by variability (see [72] for a review). For this reason more research with different experimental paradigms should examine potential moderators of gender differences in the response to infant cues. More studies are needed to understand which individual-difference factors affect implicit reactions toward infants.

Swain [73] proposed a neurological model that attempts to explain how visual and auditory baby cues might activate parental behaviors. According to the model, infant cry, appearance, touch, and smell are organized by the sensory cortex which appraises the input and interacts with subcortical structures. In this perspective, infant cues can activate appropriate behaviors by stimulating reflexes, cognitions, and alarm/emotional systems. Different stimuli can drive similar behaviors although for different reasons. Our behavioral paradigm might tap the positive valence of reflexive impulses associated with the human infant face. Further study could investigate adults' implicit reactions associated with other infant stimuli (e.g., cry).

Finally, in this study we evaluated relations between affective implicit reactions associated with baby faces and actual or ideal self-reported caregiving styles. Positive, moderate, and significant relations emerged between baby face SC-IAT scores and beliefs about ideal social parenting, but not actual parental styles (evaluated only for parents). This result is not trivial if we consider that parenting beliefs are key aspects of parenting because they are acknowledged to generate and organize parental behaviours and mediate the effectiveness of parenting [69],[74]–[76]. More data are needed to disambiguate relations between implicit affective reactions and actual child-rearing practices. In addition, the role of social desirability in self-reported parental styles requires further investigation [77].

## Conclusions

The results of this study show that human infant faces represent highly biologically relevant stimuli that capture attention and are implicitly associated with positive emotions [10],[48],[50]. Moreover these relations seem to hold across gender and parenthood status and are associated with desirable instrumental parenting beliefs. Our data further indicate that adults show individual variation in implicit dispositions toward babies. Future studies are needed to evaluate possible associations of this implicit and intuitive form of cognition with actual parental practices and with possible risk factors. Indeed, a reliable and valid association of affective implicit reactions with parental behaviours would constitute a first step in the development the SC-IAT toward baby cues as a screening instrument for infant-adult dyads at possible risk to wholesome child development.
